# A Novel Neurotoxin Gene *ar1b* Recombination Enhances the Efficiency of *Helicoverpa armigera* Nucleopolyhedrovirus as a Pesticide by Inhibiting the Host Larvae Ability to Feed and Grow

**DOI:** 10.1371/journal.pone.0135279

**Published:** 2015-08-21

**Authors:** Huan Yu, Jiao Meng, Jian Xu, Tong-xian Liu, Dun Wang

**Affiliations:** 1 State Key Laboratory of Crop Stress Biology for Arid Areas Northwest A&F University, Yangling, Shaanxi, P. R. China; 2 Key Laboratory of Applied Entomology, Northwest A&F University, Yangling, Shaanxi, P. R. China; Natural Resources Canada, CANADA

## Abstract

A recombinant *Helicoverpa armigera* nucleopolyhedrovirus (HearNPV), Ar1b-HearNPV, was constructed and identified as an improved bio-control agent of *Helicoverpa armigera* larvae. The HearNPV *polyhedrin* promoter was used to express the insect-specific neurotoxin gene, *ar1b*, which was originally isolated from the Australian funnel-web spider (*Atrax robustus*). RT-PCR and Western blotting analysis showed that both the *ar1b* transcript and protein were produced successfully in Ar1b-HearNPV-infected HzAM1 cells. In order to investigate the influence of foreign gene insertion in HearNPV, including the *ar1b* gene, chloramphenicol resistance gene, *lacZ*, kanamycin resistance gene, and the gentamicin resistance gene, two virus strains (HZ8-HearNPV and wt-HearNPV) were used as controls in the cell transfection analysis. As expected, foreign gene insertion had no impact on budded virus production and viral DNA replication. Both optical microscopy and electron microscopy observations indicated that the formation of the occlusion bodies of recombinant virus was similar to wild type virus. The Ar1b-HearNPV-infected *H*. *armigera* larvae exhibited paralysis and weight loss before dying. This recombinant virus also showed a 32.87% decrease in LT_50_ assays compared with the wild type virus. Besides, Ar1b-HearNPV also inhibited host larval growth and diet consumption. This inhibition was still significant in the older instar larvae treated with the recombinant virus. All of these positive properties of this novel recombinant HearNPV provide a further opportunity to develop this virus strain into a commercial product to control the cotton bollworm.

## Introduction

Some microorganisms such as fungi, bacteria, nematodes and viruses have been effectively used as bioinsecticides due to their host-specificity and their lack of environmental and health hazards [1, 2]. Baculoviruses, as specific pathogens for insects, are potential agents for biological pest control. Although many baculovirus species have been well characterized, only a few have been developed as commercial insecticides, such as the *Cydia pomonella* granulovirus applied on the codling moth (*Cydia pomonella*) and the *Anticarsia gemmatalis* nucleopolyhedrovirus used to control the velvet bean caterpillar (*Anticarsia gemmatalis*) [3, 4].

The cotton boll worm (*Helicoverpa armigera* (Hubner)) infects a wide range of plants and is a particularly serious pest of cotton in China. The damage to leaves and cotton bolls caused by *H*. *armigera* larvae results in dramatic losses in cotton production. The ability of *H*. *armigera* larvae to develop a strong resistance to chemical insecticides and to the *Bacillus thuringiesis* toxins used in genetically modified cotton has made the use of traditional chemical pesticides and *Bt* transgenic engineered cotton plants varieties far less effective [5–9]. The *Helicoverpa armigera* single nucleocapsid nucleopolyhedroviruses (HearNPV) is a highly infectious and selective pathogen of cotton bollworm and has been successfully developed as a commercial bio-pesticide [10]. However, like other baculoviruses, HearNPV suffers the disadvantage of having a relatively slower killing speed compared to chemical insecticides, and a lower virulence against older instar larvae [4].

Genetic engineering has been used to modify the insecticidal activity of baculovirues [11–13]. The ecdysteroid UDP-glucosyltransferase gene (*egt*) deleted recombination in *Autographa californica* multicapsid nucleopolyhedroviruses (AcMNPV) [14–16] or *Lymantria dispar* multicapsid nucleopolyhedroviruses (LdMNPV) [17] can accelerate the death of the host larvae by 11 and 33%, respectively. The insertion of insect-selective toxin expression genes has led to some very encouraging pest control results [18–24]. Laboratory studies have demonstrated that various HearNPVΔ*egt* and HearNPV-AaIT recombinants killed larvae of selected lepidopteran species from 27% to 32% faster than wild-type HearNPV [25]. When these *egt*-minus and toxin-containing recombinant HearNPV were evaluated on cotton in the field, they showed a reduced ST_50_ value of 15.3 and 26.3% compared with wt-HearNPV [26].

Our goal was to construct an appropriate insect-specific neurotoxin gene recombinant HearNPV which can increase the virulence of HearNPV, especially reducing the pest’s consumption of the host plant. Ar1b as a representative of ω-atracotoxins was originally isolated as a 7.7 kDa peptide from the venom of the Australian funnel-web spider *Atrax robustus* (GenBank accession NO. EF523495). The bacterial expressed Ar1b has been reported to be hypertoxic to armyworm (*Mythimna separate*) by injection [27]. Laboratory analysis in this study demonstrated that the *ar1b* recombinant HearNPV has a great potential to shorten the killing time on *H*. *armigera* larvae with fewer occlusion bodies (OBs) compared with wt-HearNPV. Another novel property of this recombinant HearNPV is to paralyze the larva and reduce food consumption. All of these properties of this novel recombinant virus will allow this stain to be further developed as a bioinsecticide.

## Materials and Methods

### Cells, virus, and insects

The HzAM1 cells were grown in TNM-FH medium (Sigma, USA) with 10% FBS (Gibco, USA), streptomycin (30 μg/ml), and penicillin (100 μg/ml) at 27°C incubator. The HearNPV virus was provided by Dr. Chuanxi Zhang at Zhejiang University in China. The HearNPV Bacmid HaBacHZ8 (propagated in *E*. *coli* BW25113) was constructed by Dr. Hanzhong Wang at the Wuhan Institute of Virology, Chinese Academy of Sciences [28]. A laboratory colony of cotton bollworm, *H*. *armigera*, was cultured on an artificial diet at 28±1°C and a 16-h light/8-h dark photoperiod.

### Construction of the donor plasmid

**Fig 1:**
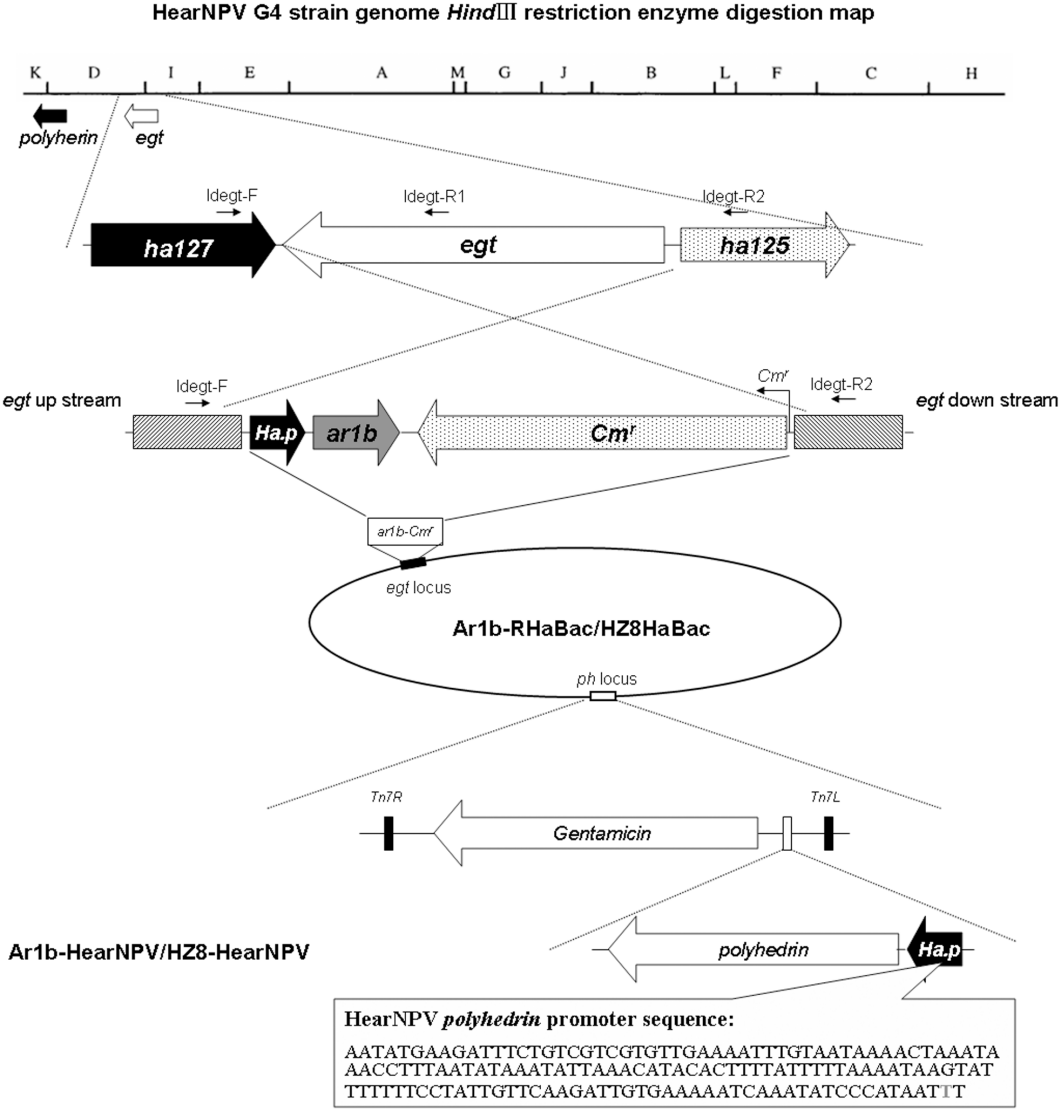
Construction of Ar1b-HearNPV and HZ8-HearNPV bacmids. A *Hind*III digestion map of the HearNPV G4 strain genome (GenBank accession number: AF271059) and schematic diagram of the *egt* replacement by *ar1b* and *cm* ^*r*^ gene is shown at the top. Correct recombination was confirmed by PCR amplification and *Hind*III digestion analysis. The primer positions are shown on the upper part of the diagram. The lower part of the figure shows the *polyhedrin* gene inserted into the Ar1b-HaBac or HZ8-HaBac by site-to-site transposition to generate Ar1b-HearNPV and HZ8-HearNPV. A 148 bp *polyhedrin* native promoter sequence is shown, in which a G was mutated into a T (in bold and gray) so as to avoid any polyhedrin translation frameshift.

The donor vectors for the *egt* locus region replacement by *ar1b* tagged with hemagglutinin (HA) and a chloramphenicol resistance gene (*Cm* ^*r*^) were constructed as follows. A 900-bp *egt* upstream homologous fragment was PCR amplified from the HearNPV-G4 genome (AF271059.1) with primer pair egt-Ff/egt-Fr (Table 1). The PCR products were then subcloned into pFastBac HTb to generate a recombinant plasmid HTb-egtF. A 1000-bp *egt* downstream homologous fragment was cloned into HTb-egtF using the primers egt-Rf/egt-Rr (Table 1) as described above to form the new vector HTb-egtF-egtR. The HearNPV *polyhedrin* promoter, Haph.p (-144nt– 4nt), was synthesized using primers Haph.p-F and Haph.p-R (Table 1) from HearNPV-G4. The PCR product was directly digested with *Pst*I and *Sal*I and was then ligated to the HTb-egtF-egtR that was again digested with the same two enzymes resulting in HTb-egtF-Haph.p-egtR. With primers Cm^r^-F and Cm^r^-R (Table 1), a 937-bp fragment containing the chloramphenicol resistance gene with the T7 promoter was PCR amplified from plasmid PKSE (constructed in Dr. Hu’s lab at the Institute of Virology, CAS). Following *Kpn*I and *Xho*I digestion, the PCR product was cloned into the plasmid HTb-egtF-Haph.p-egtR to construct the recombinant vector HTb-egtF-Haph.p-Cm^r^-egtR. The 222-bp *ar1b* gene with a HA-tag coding sequence at the 3’ end was amplified from pET 32a(+)-Ar1b [27] using primers toxin-F and toxin-R (Table 1). The toxin-encoding fragment was then cloned into HTb-egtF-Haph.p-Cm^r^-egtR to generate the final recombinant transfer vector HTb-egtF-Haph.p-Ar1b-Cm^r^-egtR (Fig 1). The inserted fragment sequences for all of those recombinant vectors were verified by DNA sequencing at each step.

**Table 1:** Primers used in this study.

Primer	Sequence^a^	Location (nt)^b^	Amplification purpose
egt-Ff	5’- AGGATCCAGTCAATGCTGCGACACGCGT -3’ (*BamH*I)	117721→117745	Primers for *egt* upstream homologous fragment amplification from HearNPV-G4 strain.
egt-Fr	5’- CGTCGACCTATTTGAATTTAGCAATAAA -3’ (*Sal*I)	118608→118628	
egt-Rf	5’- CGGTACCTACTGTATGACAATGTACACA -3’ (*Kpn*I)	120174→120194	Primers for *egt* downstream homologous fragment amplification from HearNPV-G4 strain.
egt-Rr	5’- TAAGCTTACGTGTCGTCTGCCGTCGGTA -3’ (*Hind*III)	121150←121170	
Haph.p-F	5’- TGTCGACGCCTGAGGGATTTCTGTCGTCGTGTTGA -3’ (*Sal*I)	131262→113289	Primers for *polyhedrin* promoter amplification from HearNPV-G4 strain.
Haph.p-R	5’- CCTGCAGGAATTATGGGATATTTGATTTTTC -3’ (*Pst*I)	131386←4	
Cm^r^-F	5’- AGGTACCTTCCTGTGCGACGGTTACGCCGCT -3’ (*Kpn*I)		Primers for chloramphenicol resistance gene amplification from PKSE vector.
Cm^r^-R	5’- ACTCGAGTTTAAGGGCACCAATAACTGCCTT -3’ (*Xho*I)		
toxin-F	5’- ACTGCAGATGAATACCGCAACAGGT -3’ (*Pst*I)		Primers for the insect specific toxin gene *ar1b* amplification from pET 32a(+)-Ar1b vector.
toxin-R	5’- GCTCGAG***TTA****AGCGTAATCTGGTACGTCGTATGGGTAACA*AACGCATCTTTTAAC -3’ (*Xho*I)		
Idegt-F	5’- AGCATCCCGTCCGTTGGTCAT -3’	118540→118560	Primers for homologous recombination identification in the HZ8-HaBac.
Idegt-R1	5’- CTCGTATAGACGTACCGAAGC -3’	119530←119550	
Idegt-R2	5’- GCAATAGGAGACGGGTAGTCA -3’	120250←120270	
ph-F	5’–CGGATCCATGTATACTCGTTACAGT- 3’ (*BamH*I)	4→23	Primers to amplify the CDs of HearNPV *polyhedrin*.
ph-R	5’–ACTGCAGTTAATATGCAGGACCAGT- 3’ (*Pst*I)	727←744	
pUC/M13-F	5’- CCCAGTCACGACGTTGTAAAACG -3’		Primers for site specific transposition verification.
pUC/M13-R	5’- AGCGGATAACAATTTCACACAGG -3’		
qβ-actin-F	5’- GAGAAGATCTGGCACCACACCTTCT -3’		*β-actin* specific primer for qPCR
qβ-actin-R	5’- CTCGTAGATGGGCACGGTGTGCGAC -3’		
qAr1b-F	5’- ATGAATACCGCAACAGGTTTCATTG -3’		*ar1b* specific primer for qPCR.
qAr1b-R	5’- TTTCGTTATACGGGCACGGTTGACC -3’		
qGp41-F	5’- CATCCGATTAGCGTGAACG -3’	66316←66334	*gp41* specific primer for qPCR.
qGp41-R	5’- GGGCATAACTCGGCAACAC -3’	66181→66199	

^a^ HA-tag coding sequence is in italics with a TAA stop codon in bold at the 3’ end of Ha-tag, restriction enzyme sites are underlined.

^b^ The location of each primer cited in the table was accorded to the HearNPV-G4 strain genome sequence reported in NCBI (AF271059).

### Generation of *ar1b* or *egfp* recombinant HearNPV bacmid

To generate the *ar1b* recombinant HearNPV bacmid, the fragment of egtF-Haph.p-Ar1b-Cm^r^-egtR was cut from donor plasmid HTb-egtF-Haph.p-Ar1b-Cm^r^-egtR using *BamH*I and *Hind*III. The resulting linear fragments containing the toxin gene and *Cm* ^*r*^, *ar1b* and flanking region of *egt* was gel purified and then transformed into *E*. *coli* BW25113 cells containing the HaBacHZ8-bacmid and a pKD46 plasmid to generate the homologous recombination by ECM 399 electroporation system (BTX, USA). The positive clones with chloramphenicol and kanamycin resistance were selected after 48 hours incubation at 30°C. Two pairs of primers were used for primary confirmation of the *egt* replacement in the HaBacHZ8 bacmid for the two recombinants. Primers Idegt-F and Idegt-R1 (Table 1) were used to detect the correctness of the foreign gene insertions (Fig 1). Primers Idegt-F and Idegt-R2 (Table 1), which are located on either side of *egt*, were used to confirm conjunction of the recombinant region (Fig 1). Restriction enzyme *Hind*III (TaKaRa) digestion analysis was used to verify the recombination. The digested product was separated by electrophoresis with 0.8% agarose gel at 65 V for 6h. The resulting recombinant bacmid was named Ar1b-HaBac (Fig 1).

### Site-specific transposition for polyhedrin gene reintroduction into the recombinant bacmid

To determine the effect of *ar1b* recombination in HearNPV and examination for infectivity, the *polyhedrin* (*ph*) gene was reintroduced into the *ph* locus of the Ar1b-RHaBac and HaBacHZ8-bacmid by using a site-specific transposition. First a repair donor plasmid in which the *ph* gene with its own polyhedrin promoter was constructed as follows. A 160-bp HearNPV *polyhedrin* promoter (*Ha*.*p*) with *Bst1107*I and *Bam*HI sites on the each side was synthesized in which the ATG of the polyhedrin promoter was mutated to ATT (Fig 1A). *Ha*.*p* were cut out as a *Bst1107*I/*Bam*HI fragment and ligated with pFastBac HTb which was also digested with *Bst1107*I and *Bam*HI mixture to generate a new vector Ha.p-HTb. A 741-bp *ph* gene was PCR amplified from HearNPV-G4 with primers ph-F and ph-R. The PCR product was digested with *BamH*I and *Pst*I and then ligated into vector Ha.p-HTb which was also digested by *BamH*Iand *Pst*Ito generate a recombinant plasmid renamed as Ha.p-ph-HTb.

A helper plasmid pMON7124 was isolated from DH10Bac (Life Technologies) cells and transformed into Ar1b-RHaBac/BW25113 and HaBacHZ8/BW25113 cells, respectively. Then the competent cells for Ar1b-RHaBac/BW25113 and HaBacHZ8/BW25113 with the helper plasmid were transformed with the Ha.p-ph-HTb donor plasmid which contains the *ph* gene with its own promoter. After 4 h of incubation at 37°C in 1 ml SOC medium, cells were spread onto LB agar medium containing kanamycin (50 μg/ml), gentamicin (14 μg/ml), tetracycline (10 μg/ml), Bluo-Gal (100 μg/ml) and IPTG (40 μg/ml). Plates were incubated at 37°C for 48 h; big white colonies were selected and re-streaked onto fresh plates to verify the phenotype. Ten clones were selected and bacmids were extracted from each clone. Primers pUC/M13-F and pUC/M13-R were used to confirm the insertion of the *polyhedrin*. The PCR products were sequenced for further confirmation. The *polyhedrin*-repaired recombinant bacmids were named Ar1b-HearNPV and HZ8-HearNPV, respectively (Fig 1).

### Transcription and expression analysis of *ar1b*

To make sure the *ar1b* inserted into the HearNPV genome was active, both reverse transcription PCR (RT-PCR) and western blot analysis were used to detect expression of *ar1b*. The total RNA was extracted from Ar1b-HearNPV infected HzAM1 cells (2 × 10^6^) with RNAiso plus (TAKARA) according to the manufacturer’s protocol. Briefly, the cDNA was synthesized using 1.0 μg of total RNA (as template) with the reverse transcriptase M-MLV (Thermo Fisher Scientific, USA) and an oligo(dT) anchor primer. Primers qβ-actin-F and qβ-actin-R (Table 1) were used to detect the β-actin transcription; this was selected as a reference gene. The *ar1b* transcription level was monitored by primer pairs qAr1b-F and qAr1b-R (Table 1).

Ar1b-HearNPV-infected HzAM1 cells were used to extract the total protein according to the manufacturer’s guidelines [29]. Protein samples were then separated by SDS-PAGE (15%) and electro-transferred by Trans-Blot SD semi-dry transfer cell (BIO-RAD) to the nitrocellulose membrane. Immunoblotting was performed by using standard protocols. The primary antibodies used included a monoclonal anti-actin antibody (1:10000) and a monoclonal anti-HA (1:4000), following the secondary antibodies HRP-conjugated goat anti-mouse lgG (1:5000). The proteins were visualized with an enhanced chemi-luminescence system (ECL; GE).

### Virus growth and virus DNA replication analysis of Ar1b-HearNPV

The viral growth curve analysis was performed to determine whether the virus production was affected by the *egt* deletion or the *ar1b* insertion. Transfection into HzAM1 cells (2 × 10^6^) with 1.5 μg of the bacmid DNA (Ar1b-HearNPV, HZ8-HearNPV, or wt-HearNPV) was performed using X-tremeGENE HP DNA Transfection Reagent (Roche, SUI). At each time point, the supernatant containing BVs was collected and all samples were tested for the 50% tissue culture infective dose (TCID_50_). The DNA replication curve of the recombinant virus was investigated by qPCR as described according to Li [30]. The general linear method (GLM) in SPSS 15.0 (SPSS Inc, Chicago, IL) was used to establish the difference between different virus strains [29].

### Electron microscopy observation

1.0 *μ*g each of the constructed bacmid for Ar1b-HearNPV, HZ8-HearNPV, or wt-HearNPV was transfected to 10^6^ HzAM1 cells (35-mm-diameter well), respectively. At 72 h p.i., cells were collected by centrifuging at 2000 × g for 10 min. Occlusion bodies, purified from the cadavers of *H*. *armigera* larvae that had been infected with Ar1b-HearNPV, HZ8-HearNPV, or wt-HearNPV, were washed and re-suspended in 100 μl of 2% agarose. The OBs-containing agarose pellets were washed in double sterile water and sliced into 2 mm cubes. Both the cells and OBs were fixed in 2.5% glutaraldehyde overnight, and then dehydrated, embedded, sectioned, and stained as described according to Li [30]. Samples were examined using an Hitachi HT7700 TEM with an accelerating voltage of 80 kV.

### Virus propagation and purification

To enlarge the scale of virus production, 2 μl of each BV supernatant (from HzAM1 cell infection of Ar1b-HearNPV, HZ8-HearNPV or wt-HearNPV) with a titer of 10^5^ TCID_50_ ml^-1^ was injected into third-instar *H*. *armigera* larva. The negative control was the TMN-FH cell culture medium. Infected larvae were reared individually in 24-well insect culture boxes and monitored daily until all larvae had either pupated or died. The insect larvae cadavers that died from virus-infection were collected in a stout plastic bag and thoroughly macerated in 0.1% SDS. The pellets containing the occlusion bodies and insect tissue debris were filtered through 4 layers of muslin and then centrifuged at 3000×*g* for 30 min. The precipitate was re-suspended in 10 volumes of TE buffer and was rotated for 1 h. 10 ml of the thoroughly mixed and re-suspended OBs in TE buffer was then placed on a sucrose step gradient (6 ml of 90% sucrose, 6 ml 75% sucrose and 6 ml 50% sucrose) and centrifuged at 90,000×*g* for 1 h. OBs were collected at the interface between the 50% and 75% sucrose layers, diluted with 10 volumes of TE buffer, centrifuged for 15 min at 3000×*g*, and re-suspended in a TE buffer or double sterile water. Appropriate dosages for bioassays were prepared in the same diluted and quantified using a hemocytometer.

### Lethal dose and survival time of infected *H*. *armigera* larvae

The LD_50_ (median lethal dose) and LT_50_ (median lethal time) values of the HearNPV variants were tested by a droplet-feeding bioassay [20, 31]. Third instar larvae of *H*. *armigera* were fed the suspension of OBs (7 to 2000 OBs/larva) mixed with 5% sucrose and 3% blue food color (Shaanxi TOP Pharm Chemical Co., Ltd, China). Larvae that ingested the whole droplet were transferred individually into 24-well insect culture boxes with food. Dead larvae were recorded and removed daily until all larvae died or pupated.

LD_50_ values were tested for three viruses (Ar1b-HearNPV, HZ8-HearNPV, or wt-HearNPV). Five virus dosage levels (7, 15, 30, 60, and 120 OBs per larva) were used for each virus. Each dose was tested using 30 3rd-instar larvae. LT_50_ values were tested with LD_90_ dose for 3rd instar larvae. All tests were repeated three times. The data values were analyzed using a probit analysis via SPSS and compared by paired-sample t-tests [32].

### Artificial diet consumption of virus-infected *H*. *armigera* larvae

Another thirty 2nd, 3rd, and 4th instar *H*. *armigera* larvae were fed with each virus variant with a LD_90_ for laboratory artificial diet consumption analysis. These larvae were transferred to sufficient artificial diet plugs every 12 h and cultured individually in 24-well insect culture boxes. Diet consumption was calculated by a weight change of the supplied diet. Control larvae were fed on virus-free diet plugs. The consumption of tested larvae for various HearNPVs was compared by GLM in SPSS.

### Weight gain assay of larvae

Thirty larvae were selected to analyze the daily weight gain and were treated as described above. The weights of all tested larvae were recorded daily at 9 am until the larvae died or pupated. The daily weight gains of tested larvae for various HearNPVs starins were compared by GLM in SPSS.

## Results

### Construction of *ar1b* recombinant HearNPV

**Fig 2:**
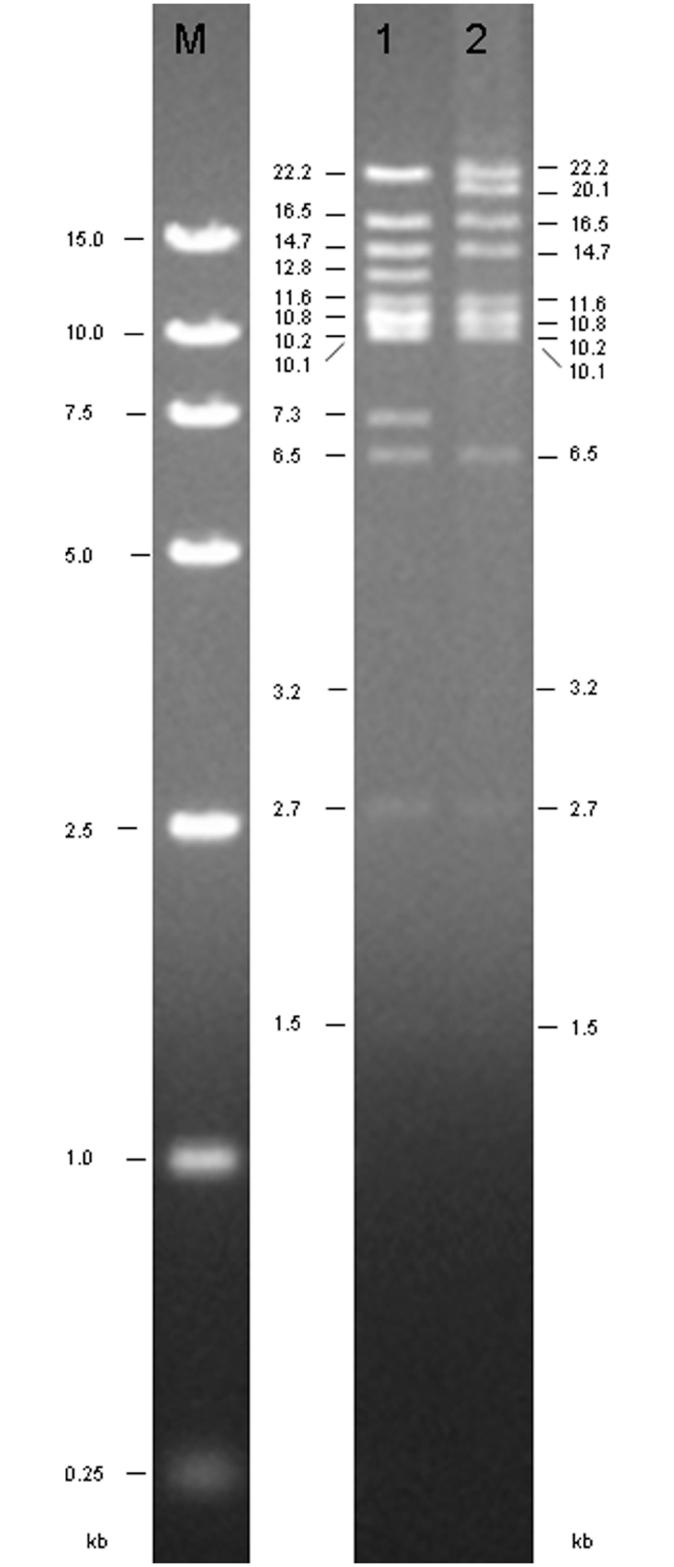
*Hind*III restriction enzyme digestion identification of *ar1b* recombination. The size of each band is indicated beside each lane (kb): Lane M: DNA ladder 15,000. Lane 1: HZ8-HaBac bacmid DNA digested by *Hind*III; Lane 2: Ar1b-HaBac bacmid DNA digested by *Hind*III.

The structure of the donor plasmid HTb-egtF-Haph.p-Cm^r^-egtR which contains the *ar1b* gene, and the *egt* locus in the HearNPV genome are presented in Fig 1. A 1750 bp fragment was detected from HZ8-HaBac using primers IdegtF and IdegtR2, while a 1500 bp fragment was detected from Ar1b-RHaBac with the same primers (data not shown). Instead of a 1000 bp fragment amplified from HZ8-HaBac with primers IdegtF and IdegtR1, no products were obtained from Ar1b-RHaBac because the *egt* was deleted by the recombination (data not shown). The *Hind*III digestion profile further confirmed the replacement of *egt* with *ar1b* and *Cm* ^*r*^ in the HearNPV bacmid. Because there is a *Hind*III site contained in *egt*, the wild type 12.8-kbp D *Hind*III-fragment and 7.3-kbp I *Hind*III-fragment in HZ8-HaBAC digested products were combined into a new 20.1 kb *Hind*III-fragment in Ar1b-RHaBac by recombination (Fig 2). These results demonstrate that the *egt* gene in the HaBacHZ8-bacmid was successfully replaced by *ar1b* and *Cm* ^*r*^ genes. The *polyhedrin* gene with its endogenous promoter was repaired into the *ph* locus of Ar1b-RHaBac and HaBacHZ8 and confirmed by PCR analysis with pucM13 primers, respectively. Two fragments of 3170 bp were amplified from Ar1b-HearNPV and HZ8-HearNPV, and a 300 bp fragment was amplified from the HaBacHZ8-bacmid, (data not shown).

### Occlusion bodies were detected in Ar1b-HearNPV transfected cells

**Fig 3:**
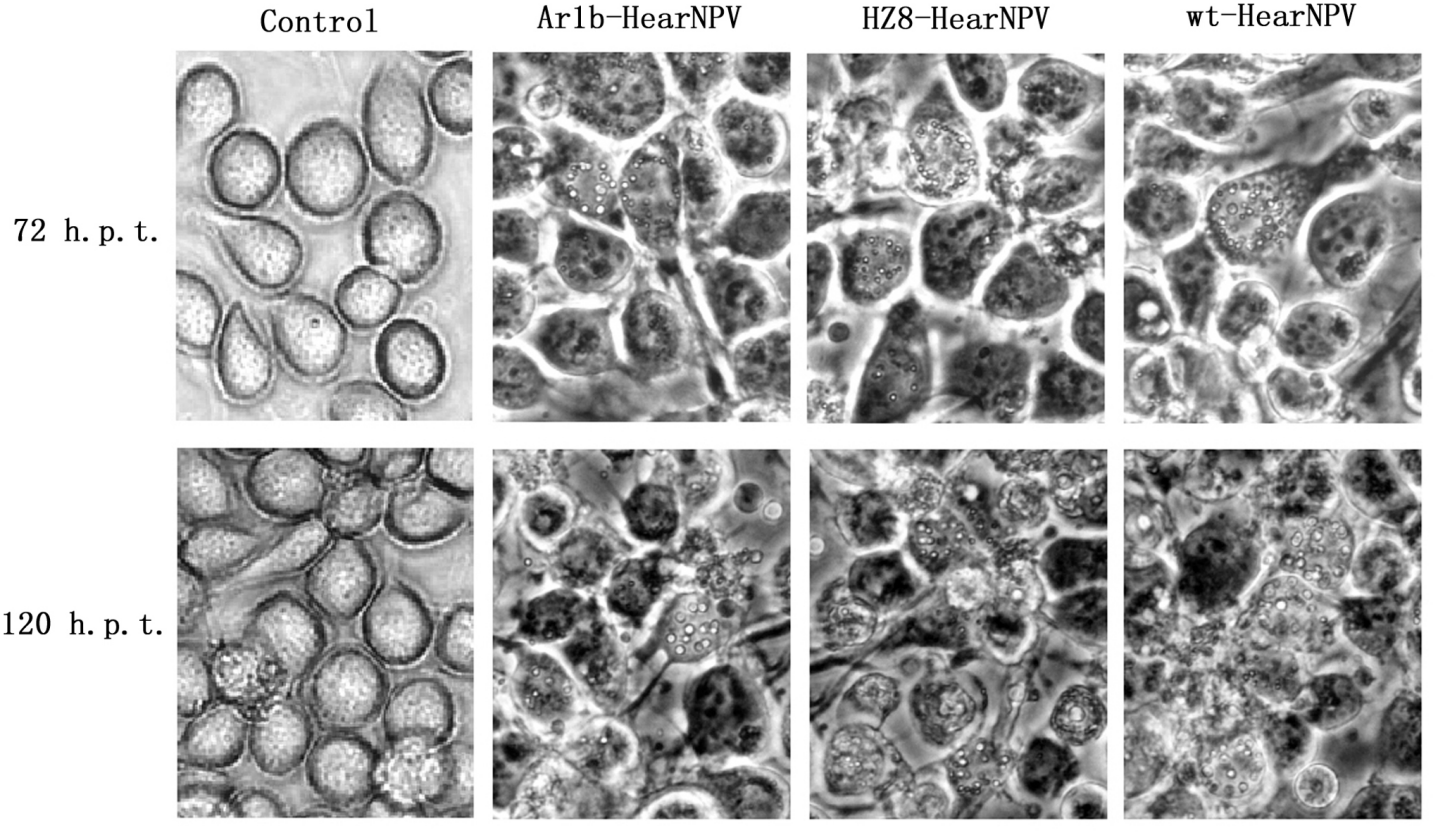
Microscopy observation of virus-transfected HzAM1 cells. The formation of occlusion bodies in Ar1b-HearNPV, HZ8-HearNPV, or wt-HearNPV infected cells at 72 and 96 h.p.t. No OBs were detected in the mock cells.

In order to investigate the effect of the *ar1b* insertion on virus infection and OBs formation, HzAM1 cells were transfected with Ar1b-HearNPV and HZ8-HearNPV, and these cells were monitored by microscopy. A similar number of Ar1b-HearNPV and HZ8-HearNPV transfected cells were observed to contain polyhedra at 72 h post transfection (p.i.). At 120 h p.i., both viruses showed a large increase in the number of transfected cells (Fig 3). The results confirmed that the insertion of *ar1b* and *Cm* ^*r*^ genes did not affect viral occlusion body formation in HzAM1 cells.

### Analysis of recombinant gene transcription and expression

**Fig 4:**
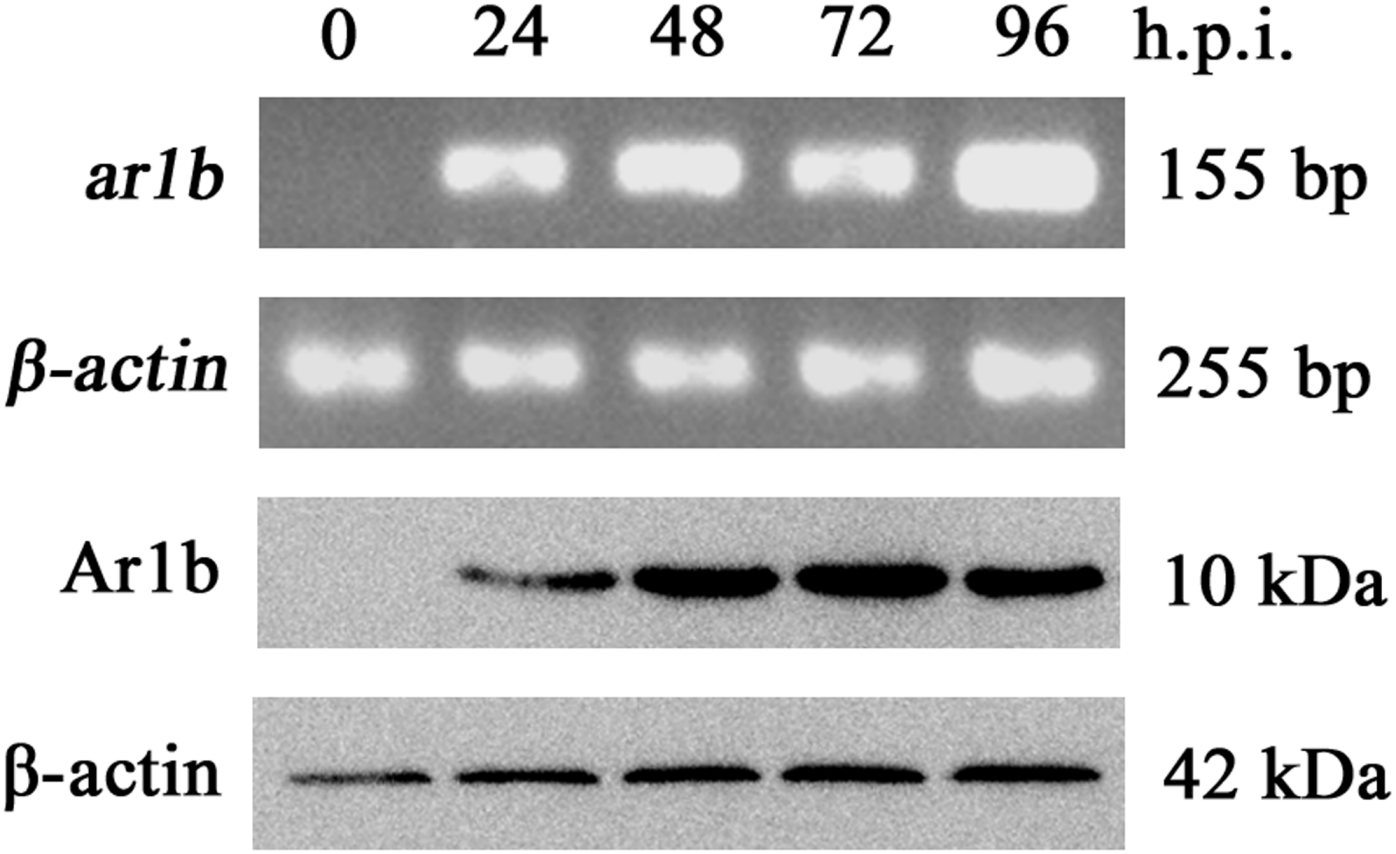
Transcription and expression verification of *ar1b* in Ar1b-HearNPV infected HzAM1 cells. The upper part of the figure shows the transcriptional analysis of *ar1b* in infected HzAM1 cells. RT-PCR was performed and the PCR products underwent electrophoresis in 1.0% agarose gel. *Β-actin* was selected as the reference gene. The lower part in the figure shows the immunoblot analysis of Ar1b in Ar1b-HearNPV-infected HzAM1 cells. Protein samples were harvested and separated by 15% SDS-PAGE. Anti-HA monoclonal antibodies were used as the primary antibody to show the Ar1b protein expression bands.

The recombinant virus was tested by reverse quantitative PCR for *ar1b* gene transcription using total RNA purified from Ar1b-HearNPV-infected HzAM1 cells or larvae at various time points after infection. A single band (bp) of *ar1b* specific product was exhibited at 24–96 h.p.i. from virus infected cells (Fig 4). No signal was detected from the RNA isolated from Ar1b-HearNPV infected cells at 0 h.p.i. (Fig 4). These results suggest that *ar1b* transcription occurred under the control of the HearNPV *polyhedrin* promoter, and expression began at 24 h.p.i.

To investigate the expression of the Ar1b-HA fusion proteins in Ar1b-HearNPV-infected HzAM1 cells, anti-HA antibodies were used in western blot analysis. A specific 10 kDa band was detected at 24–96 h.p.i. in Ar1b-HearNPV-infected cells, which is consistent with our prediction (Fig 4). No specific immunoreactive band was detected from the 0 h.p.i Ar1b-HearNPV-infected cells (Fig 4). These results show that Ar1b was successfully expressed from Ar1b-HearNPV-infected cells.

### Viral growth and viral DNA replication analysis

**Fig 5:**
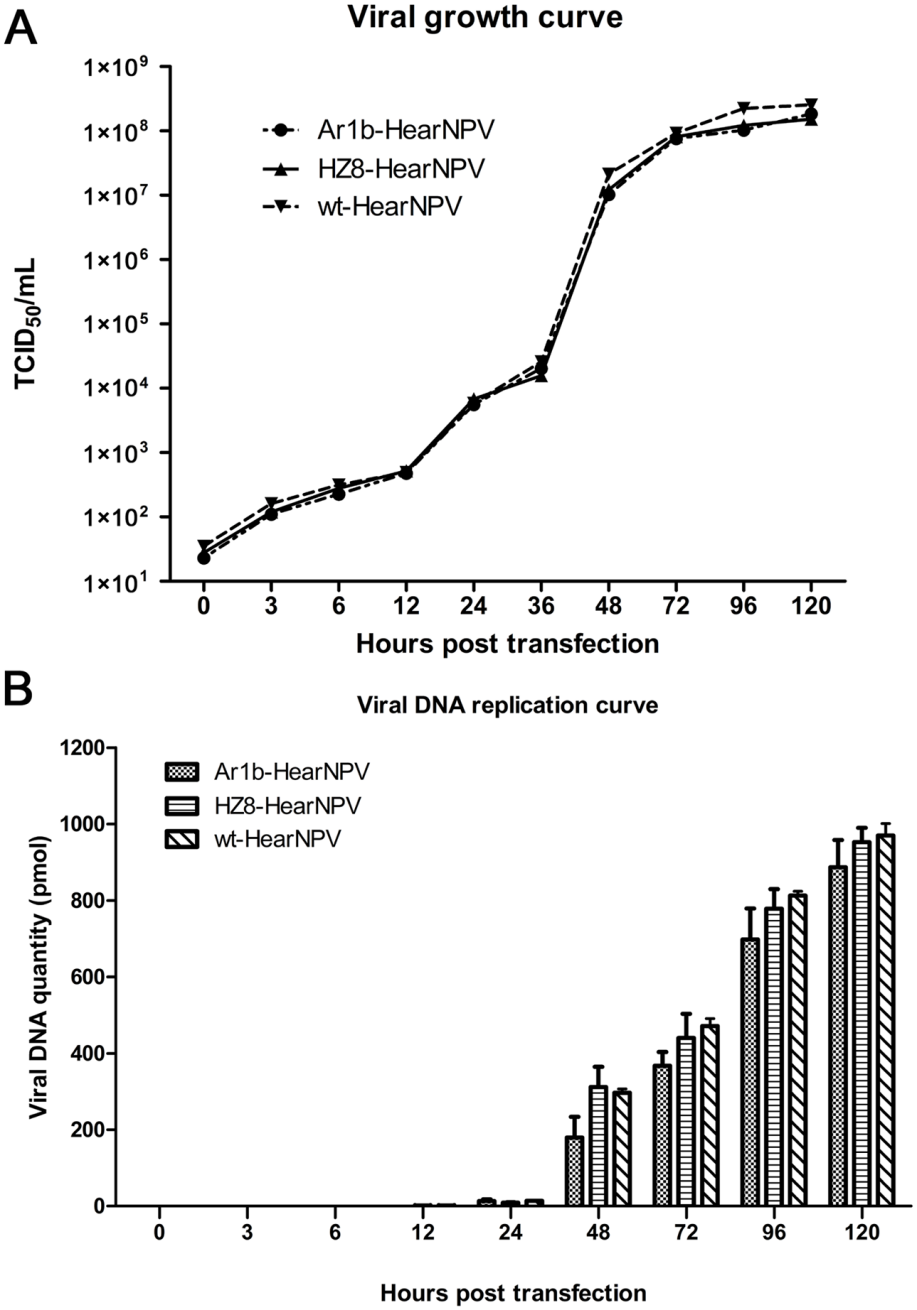
Virus growth and viral DNA replication analysis curves. A: Virus growth curves as determined by TCID_50_ endpoint dilution assays. HzAM1 cells were transfected with Ar1b-HearNPV, HZ8-HearNPV, or wt-HearNPV DNA. B: Real-time PCR analysis of viral DNA replication. Three biological replicates assays for each virus and three technical replicates for each biological replication were performed. The error bars represent the standard deviations.

To further assess the effect of Ar1b insertion on virus production, a one-step virus growth curve analysis was performed. The Ar1b-HearNPV, HZ8-HearNPV, or wt-HearNPV transfected cells revealed a similar steady increase in virus production (Fig 5A). The virus transfection growth curve indicated that Ar1b-HearNPV and HZ8-HearNPV can normally propagate in cultured HzAM1 cells. The OBs assembly and additional details were later observed by electron microscopic analysis as described below.

To quantify viral DNA replication in HzAM1 cells, the qPCR analysis was performed. The results indicate that DNA synthesis by Ar1b-HearNPV is similar to HZ8-HearNPV and wt-HearNPV (Fig 5B). As expected, Ar1b-HearNPV DNA transfected cells showed similar viral DNA copies compared to the control virus HZ8-HearNPV (F = 8.73, d.f. = 2, 4; *P* = 0.0564) or wt-HearNPV (F = 8.88, d.f. = 2, 4; *P* = 0.0601) at each selected time point (Fig 5B). The DNA replication analysis showed that the recombination of *ar1b* and *Cm* ^*r*^ genes did not affect viral DNA replication in HzAM1 cells.

### *Egt* deletion and foreign genes insertion do not affect the OBs assembling

**Fig 6:**
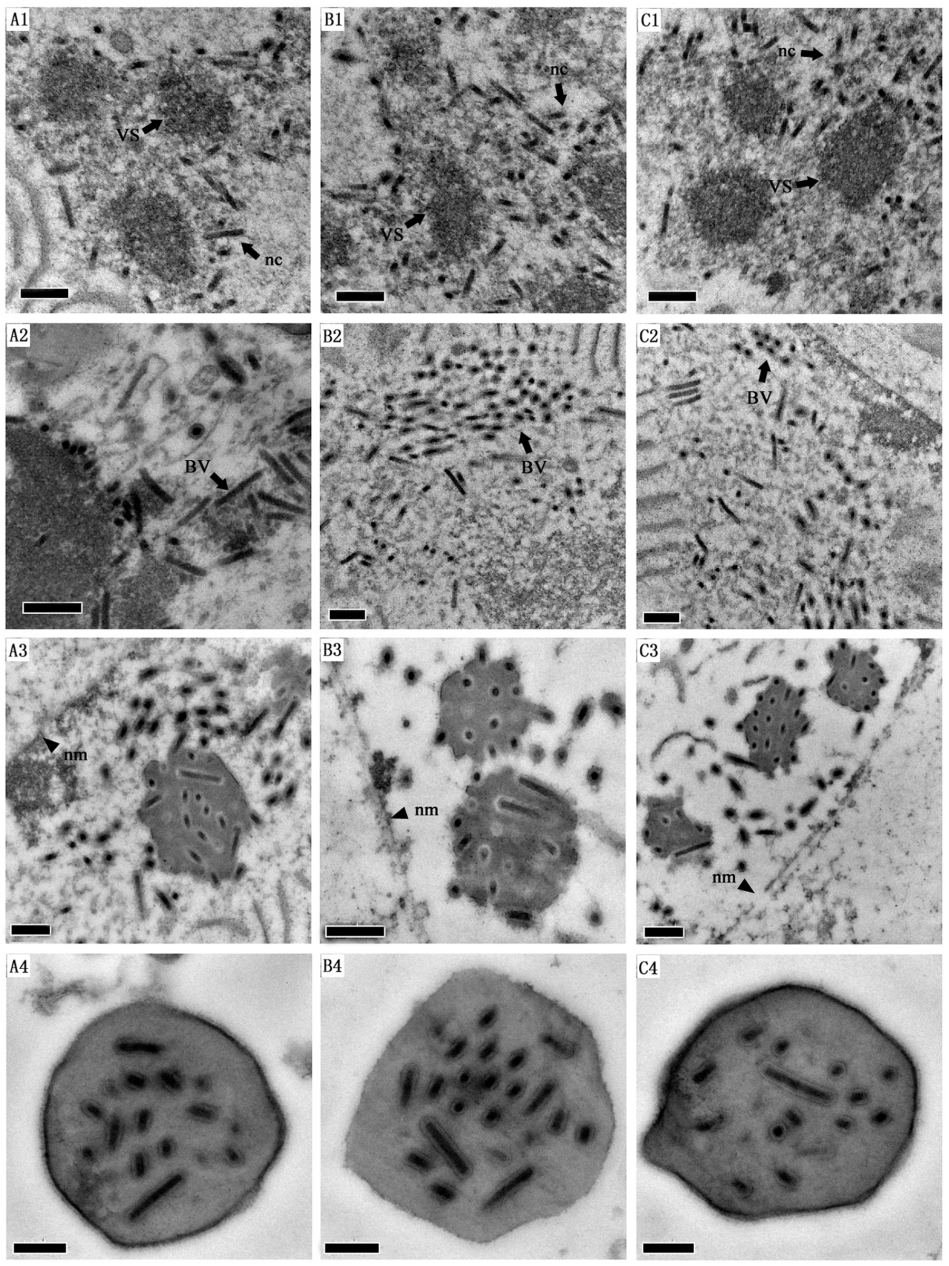
Electron microscopic analysis. HzAM1 cells were transfected with Ar1b-HearNPV (A1, A2 and A3), HZ8-HearNPV (B1, B2 and B3) or wt-HearNPV (C1, C2 and C3) DNA, and cells were fixed at 72 h.p.i. and processed for observation. OBs purified from the Ar1b-HearNPV, HZ8-HearNPV or wt-HearNPV infected larvae were observed and are shown in A4, B4 and C4, respectively. (A1, B1 and C1) Electron dense nucleocapsids (Nc, black arrows) in the virogenic stroma (VS) infected cells. (A2, B2 and C2) Bundles of nucleocapsids transformed from virogenic stroma to nuclear membrane (nm) in nucleus. (A3, B3 and C3) Polyhedra with embedded virions normally in infected cells. (A4, B4 and C4) Purified polyhedra from Ar1b-HearNPV, HZ8-HearNPV and wt-HearNPV, respectively. Scale bar, 200 nm.

Sections of virus-transfected cells were examined by electron microscopy for further analysis. An electron-dense, baculovirus-induced structure was observed in the virogenic stroma in Ar1b-HearNPV, HZ8-HearNPV and wt-HearNPV transfected cells, respectively (Fig 6A1, 6B1 and 6C1). Numerous nucleocapsids were aligned (Fig 6A2, 6B2 and 6C2) and bundles of them were prior to being occluded in the developing occlusion bodies (Fig 6A3, 6B3 and 6C3). Polyhedra were observed in the nuclei of Ar1b-HearNPV, HZ8-HearNPV, and wt-HearNPV DNA transfected cells. The shapes and sizes of the OBs from Ar1b-HearNPV transfected larvae (Fig 6A4) are similar to those in HZ8-HearNPV transfected (Fig 6B4) and wt-HearNPV cells (Fig 6C4). Together, these data indicate that the deletion of *egt* and the insertion of *ar1b* and *Cm* ^*r*^ did not affect the assembly of OBs in host cells or larvae.

### Biological activities in laboratory bioassays

**Fig 7:**
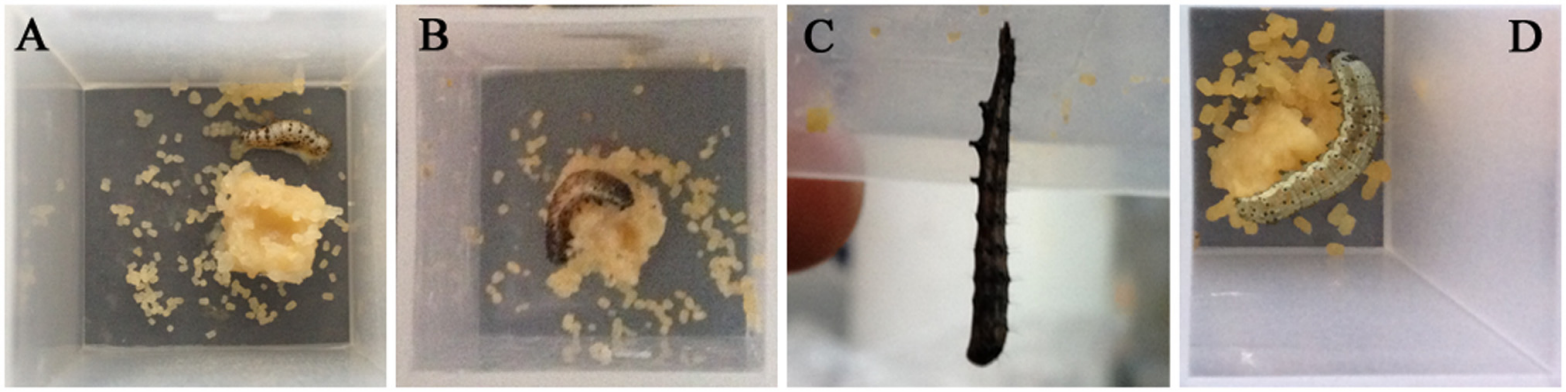
Appearance of *H*. *armigera* larvae that died from virus infection. (A) Larvae that died from Ar1b-HearNPV infection. (B) Larvae that died from HZ8-HearNPV infection. The larva body became soft and liquefied. (C) Larvae that died from wt-HearNPV infection. (D) A healthy uninfected larva fed with artificial diet in the laboratory.

The biological activity of Ar1b-HearNPV was determined through bioassay of *H*. *armigera* larvae. The LD_50_ value of Ar1b-HearNPV was slightly lower but not significantly different from the value of HZ8-HearNPV-infected larvae (Table 2). As we expected, the Ar1b-HearNPV exhibited a much lower lethal time compared to HZ8-HearNPV or wt-HearNPV (Table 3). The Ar1b-HearNPV reduced the middle lethal time by more than one day compared with wt-HearNPV (Table 3). An even greater reduction was shown in LT_90_ values, which show almost a 4 day reduction compared with wt-HearNPV. The pathological symptoms of cotton boll worms infected by Ar1b-HearNPV, HZ8-HearNPV and wt-HearNPV were also obviously different. Larvae infected with Ar1b-HearNPV showed the typical symptoms of paralysis and flaccid appearance (Fig 7A) compared with mock-infected healthy control larvae (Fig 7D). The HZ8-HearNPV-infected larvae exhibited a normal baculovirus-infection death (Fig 7B) as did wt-HearNPV-infected larvae (Fig 7C). All of these results indicate that Ar1b recombinant baculovirus presents a stronger virulence against *H*. *armigera* than does wild type and Bacmid-repaired HearNPV.

**Table 2:** Median lethal dose (LD_50_) and 90% lethal dose (LD_90_) with 90% confidence limits of *H*. *armigera* fed with Ar1b-HearNPV, HZ8-HearNPV and wt-HearNPV.

Treatment	LD_50_ (OBs/larva) (90% CL)^a^	LD_90_ (OBs/larva) (90% CL)^b^	Equation^c^	χ^2^
Ar1b-HearNPV	20.4 (16.9–240.6)	650.4 (339.0–1247.9)	y = 2.6599x − 3.2889	6.10
HZ8-HearNPV	25.1 (20.6–30.7)	1740.4 (691.5–4380.5)	y = 2.1733 x − 1.9698	2.58
wt-HearNPV	25.3 (20.9–30.4)	1633.0 (542.9–2800.6)	y = 2.3786 x +5.9284	5.80

^a, b^ Lethal doses were determined by probit in SPSS 15.0.

^c^ The intercepts of the equations of log (dose)—mortality (probit) were calculated based on the slopes and LD_50_ given by SPSS 15.0.

**Table 3:** Median lethal time (LT_50_) and 90% lethal time (LT_90_) with 95% confidence limits of *Helicoverpa armigera* fed with Ar1b-HearNPV, HZ8-HearNPV and wt-HearNPV.

Treatments	LT_50_ (Day post infection) (90% CL)^a^	LT_90_ (Day post infection) (90% CL)^b^	Equation^c^	χ^2^
Ar1b-HearNPV	4.35 (3.92–4.78)	6.58 (5.76–7.03)	Y = 6.843x-5.319	5.07
HZ8-HearNPV	6.40 (5.74–7.43)	10.51 (9.39–11.64)	Y = 6.137x-5.352	2.39
wt-HearNPV	6.48 (5.76–7.21)	11.21 (10.02–12.40)	Y = 6.142x-5.232	3.99

^a, b^ Lethal times were determined by probit analysis in SPSS 15.0.

^c^ The intercepts of the equations of log (dose)–mortality (probit) were calculated based on the slopes and LD_50_ given by SPSS 15.0.

### Survival analysis of *H*. *armigera* larvae treated with different virus dosages

**Fig 8:**
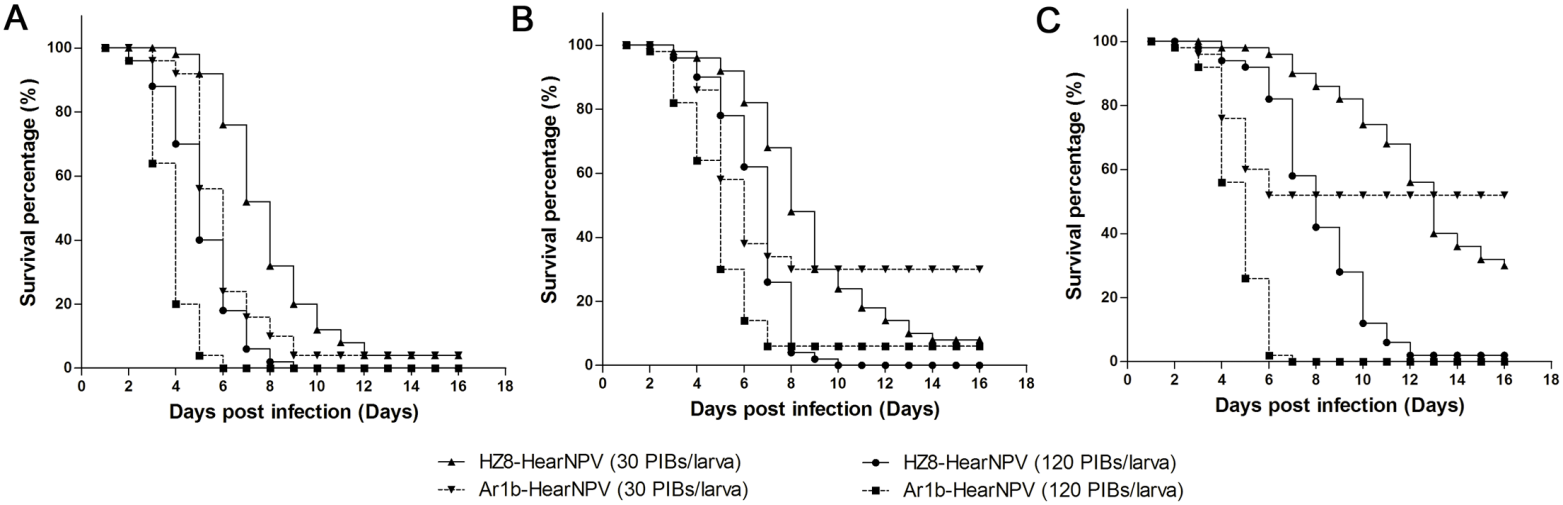
Survival analysis of *H*. *armigera* larvae treated with two different dosages (30 OBs and 120 OBs per larva) of Ar1b-HearNPV and HZ8-HearNPV. (A) Second instar larvae fed with the two dosages of virus. (B) Third instar larvae fed with the two dosages of virus. (C) Forth instar larvae fed with the two dosages of virus.

Larvae in 2nd, 3rd or 4th instars were fed with two dosages of virus, 30 OBs and 120 OBs per larva. Under the high dose treatments, both Ar1b-HearNPV and HZ8-HearNPV led to almost complete mortality in the different instars treatments. The Ar1b-HearNPV-infected 2nd instar larvae started to die at 2 days post infection (d.p.i.), which was 2 days earlier than HZ8-HearNPV-infected 2nd instar infected larvae (Fig 8A). The number of 3rd instar larvae that survived the infection with the recombinant virus started to decrease at 2 d.p.i. and reached a minimum at 7 d.p.i. (Fig 8B). It took 3 days more for HZ8-HearNPV-infected 3rd larvae to reach a minimum survival number (Fig 8B), which supports our lethal time test results described above. For the 4th instar larvae treatment groups, the Ar1b-HearNPV infected larvae showed a minimum survival rate at 6 d.p.i, which was 5 days prior to HZ8-HearNPV-infected larvae (Fig 8C). At lower dose treatments, 2nd instar larvae infected with Ar1b-HearNPV showed similar survival numbers than larvae infected with HZ8-HearNPV infected larvae, but the terminal survival percentage was reached 3 days earlier for Ar1b-HearNPV (Fig 8A). Interestingly, more larvae survived from a lower dose of Ar1b-HearNPV than HZ8-HearNPV in 3rd and 4th instars treatments. However, HZ8-HearNPV still required 3 to 5 days more to reach the terminal survival percentage (Fig 8B and 8C). Taken together, these results indicated that Ar1b-HearNPV was competent to kill the larvae within a shorter time than HZ8-HearNPV, regardless of whether the larvae were treated with the lower or higher dosage.

### Daily diet consumption analysis

**Fig 9:**
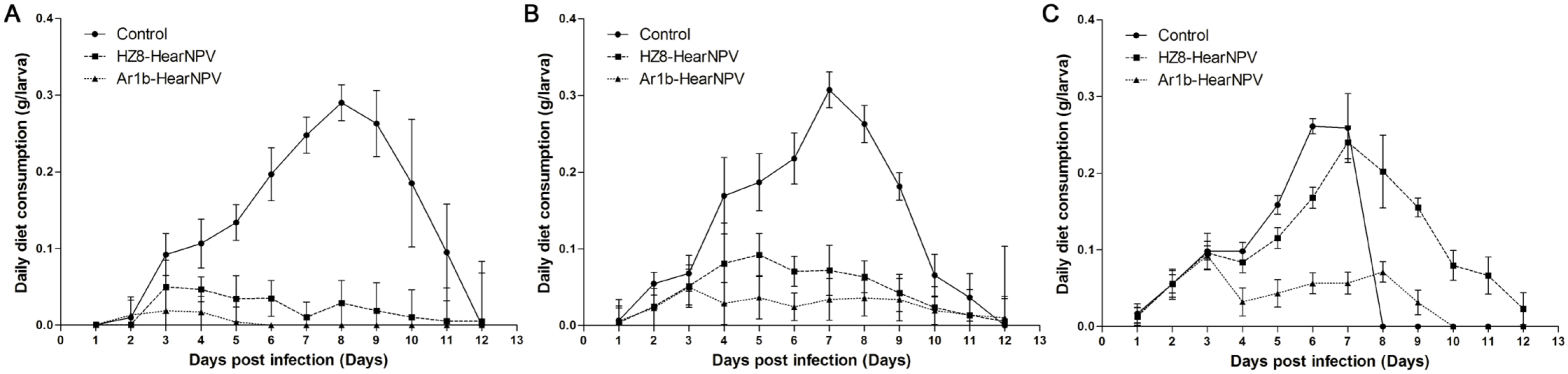
Daily diet consumption of *H*. *armigera* second, third and forth instar larvae. The daily diet consumption ± the standard deviation in virally infected second (A), third (B) and fourth (C) instar larvae are shown.

*H*. *armigera* larvae in 2nd, 3rd or 4th instar stage were tested for the consumption of an artificial diet every day. The control group larvae consumed much more food than did HZ8-HearNPV (F = 76.3, d.f. = 1, 696; *P*<0.0001) or Ar1b-HearNPV (F = 104.8, d.f. = 1, 696; *P*<0.0001) treated group larvae (Fig 9A). A significant difference was also found between the recombinant virus infected larvae and the control virus infected larvae (F = 4.8, d.f. = 1, 696; *P*<0.03) (Fig 9A). In the 3rd instar larvae treatment groups, the significant diet consumption inhibiting effects of the HZ8-HearNPV virus-infection (F = 55.3, d.f. = 1, 696; *P*<0.0001) and Ar1b-HearNPV-infection (F = 82.1, d.f. = 1, 696; *P*<0.0001) began at 4 d.p.i. compared with the mock-infected control larvae group (Fig 9B). Ar1b-HearNPV also showed stronger inhibiting effects compared with HZ8-HearNPV on 3rd instar larvae at 4 d.p.i. (F = 39.7, d.f. = 1, 696; *P*<0.0001) (Fig 9B). For the 4th instar larvae treatment groups, the recombinant virus-fed larvae started to decrease their feeding at 4 d.p.i. and the food consumption quantity was significantly lower than in the HZ8-HearNPV group larvae (F = 133.5, d.f. = 1, 696; *P*<0.0001) or healthy larvae (F = 302.4, d.f. = 1, 696; *P*<0.0001) (Fig 9C). The control group larvae pupated at 8 d.p.i. and ceased feeding, while the wild type virus-infected larvae continued growing and consumed even more food than the control group larvae (Fig 9C). These results indicate that there was a significant inhibiting effect on diet consumption in Ar1b-HearNPV-infected larvae compared with the control virus-infected larvae.

### Larval daily cumulative weight gain comparison

**Fig 10:**
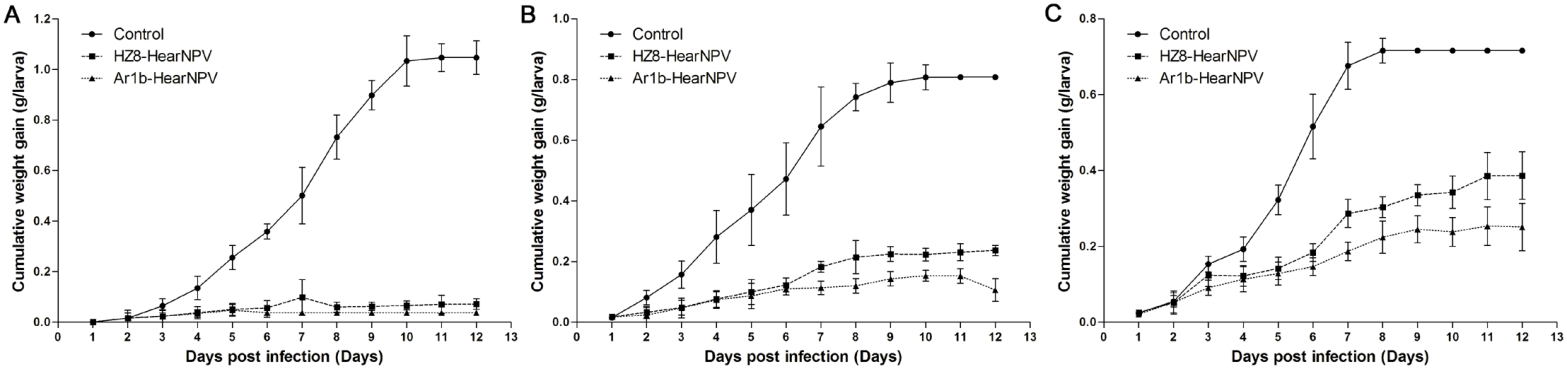
Cumulative weight gain of *H*. *armigera* second, third and fourth instar larvae. The daily cumulative weight gains ± the standard deviation in virally infected second (A), third (B) and fourth (C) instar larvae are shown.

In order to monitor the larval growth after feeding with recombinant virus or control virus, the larval daily cumulative weight gain was analysed on 2nd, 3rd and 4th instar larvae. The results show that virus infection can inhibit the 2nd instar larval growth, and there was lower weight gain in Ar1b-HearNPV infected larvae than in HZ8-HearNPV-infected larvae, and this was with a statistically significant difference (F = 4.9, d.f. = 1, 696; *P*<0.03) (Fig 10A). The 3rd instar larvae in the control group gained the most in weight, nearly 4 times more than HZ8-HearNPV group larvae (F = 604.2, d.f. = 1, 696; *P*<0.0001) and 8 times more than Ar1b-HearNPV larvae (F = 962.1, d.f. = 1, 696; *P*<0.0001) (Fig 10B). The weight of Ar1b-HearNPV-infected larvae was significantly lower than HZ8-HearNPV-infected larvae from 8 d.p.i. (F = 269,6, d.f. = 1, 696; *P*<0.0001) (Fig 10B). Clear growth inhibition was found in the 4th instar larvae treatment groups. Fourth instar control larvae gained almost 2 times more weight than HZ8-HearNPV-infected larvae (F = 323. 1, d.f. = 1, 696; *P*<0.0001) and 3 times more than recombinant virus-infected larvae (F = 485.4, d.f. = 1, 696; *P*<0.0001) (Fig 10C). Even in 4th instar larvae, Ar1b-HearNPV exhibited a significantly greater ability to cease larva growth (F = 284.5, d.f. = 1, 696; *P<*0.0001) (Fig 10C). These results indicate that Ar1b-HearNPV was able to inhibit the growth of different instar larvae, but this inhibition is weaker in 4th instar larvae compared to 2nd or 3rd instar larvae.

## Discussion

The *egt* gene was first identified in AcMNPV and is verified to alter host ecdysteroid levels prior for virus replication [14]. Deleting the *egt* gene from AcMNPV speeds up the progression to death in the host compared with a wild type virus [15]. The *egt* gene had been identified in HearNPV and was characterized [33]. In an effort to generate a HearNPV strain with improved properties, we replaced the *egt* gene with a neurotoxin gene and analyzed its biological activities.

Many promoters have been used to express foreign genes in the recombinant baculovirus construct, including the *ie-1* promoter, *p10* promoter, *polyhedrin* promoter, as well as the *p6*.*9* promoter [19, 20, 34–37]. It has been reported that different promoters lead to different insecticidal effects [32, 38]. *Polyhedrin* promoter as a strong, late promoter, has been used for expression of a foreign gene in many commercial vectors, such as pFast Bac plasmids (Invitrogen) and pIZ/V5-His plasmids (Invitrogen). In order to ensure a high level expression of the *Ar1b* neurotoxin gene, we selected the HearNPV *polyhedrin* promoter in our study.

One of the most important properties we needed this recombinant virus to improve was the virulence. Most recombinant viruses show satisfactory reduction of middle lethal dosages or middle lethal times [18, 21, 23, 24, 39–43]. The recombinant virus in this study also exhibited a great reduction in the LT_50_ value compared to wild type virus (32% reduction). Larvae infected by high dosages of Ar1b-HearNPV lead to greater mortality than WT-HearNPV. At low dosage treatments, the Ar1b-HearNPV-infection showed a lower mortality than WT-HearNPV in the fourth instar larvae (Fig 8). A similar lower mortality was shown in *Trichoplusia ni* larvae infected with a lower dosage of a scorpion toxin (LqhIT2) recombinant AcMNPV compared with wild type AcMNPV [38]. This may be due to the *egt* deletion mutants not have enough time to produce enough viruses to kill the older host larvae.

Our Ar1b-expressing recombinant HearNPV provoked symptoms of paralysis and flaccidity in infected larvae, especially one to two days prior to larva death (Fig 7A). A similar paralysis phenomenon was induced in AaIT-AcMNPV-infected *Heliothis virescens* larvae and they usually fell off the plant [39]. As we have described above, one of our aims was to construct a new virus mutant which could reduce larva consumption of the host plant. It was reported that the juvenile hormone esterase (JHE) gene recombinant AcMNPV would lead to 40–50% reduction on diet consumption in the inoculated neonate *Manduca sexta* larvae compared to wild type AcMNPV-infected larvae [44]. However, the feeding inhibition ability of JHE recombinant AcMNPV in older instar larvae is still unknown [44]. While, considerable feeding inhibition ability was shown in Ar1b-HearNPV, even in the fourth instar larvae (Fig 9). On the other hand, the larvae infected by Ar1b-HearNPV had a significant reduction in weight gain compared with HZ8-HearNPV infected larvae (Fig 10). In agreement with our results, *S*. *frugiperda* larvae infected with an *egt* gene deletion AcMNPV strain [15], and *Lymantria dispar* larvae infected with an *egt* deleted LdMNPV [17] gained less weight than larvae infected with wild-type virus. All of these improved properties suggest that application of this Ar1b-expressing HearNPV has potential to provide more effective protection of the cotton than the wild-type HearNPV. In addition, we suggest *ar1b* can be a candidate for the engineering of other baculovirus recombinant species.

The Asian cotton bollworm (*H*. *armigera*), which causes considerable economic loss to cotton and many vegetables, is an important polyphagous pest in many countries [45, 46]. The HearNPV has been registered extensively for use on cotton fields as a bioinsecticide in China [47–48]. In an effort to develop NPV insecticides for *H*. *armigera* control, scientists have isolated NPV in USA, Kenya, Australia, and China from diseased *H*. *armigera* larvae to select a candidate isolate for controlling cotton bollworm [49–54]. Several engineering methods that could improve the efficacy of HearNPV have been reported [25]. There also is the potential for recombinant HearNPV to improve the pesticidal efficiency substantially by using new neurotoxin genes from different arthropod species. Our bioassay results implied that *ar1b* insertion into the HearNPV genome could improve the insecticidal activity of HearNPV and *ar1b* could be a probable candidate for other recombinant virus species construction.
